# Synthesis of Poly (Citric Acid-Co-Glycerol) and Its Application as an Inhibitor of CaCO_3_ Deposition

**DOI:** 10.3390/ma12223800

**Published:** 2019-11-19

**Authors:** Hala Zahlan, Waseem Sharaf Saeed, Radwan Alrasheed, Naser M. Alandes, Taieb Aouak

**Affiliations:** 1 Chemistry Department, College of Science, King Saud University, P.O. Box 2455, Riyadh 11451, Saudi Arabia; wsaeed@ksu.edu.sa (W.S.S.); nandis@ksu.edu.sa (N.M.A.); 2 National Center for Water Technology, King Abdulaziz City for Science and Technology, Riyadh 11442, Saudi Arabia; ralrasheed@kacst.edu.sa

**Keywords:** citric acid-co-glycerol hyperbranched polyesters, calcium carbonate, crystallization, antiscalant, conductivity, static scale inhibition

## Abstract

This investigation determined a feasible route to prepare hyperbranched polyesters involving citric acid (CA) and glycerol (GLC) monomers (CA-co-GLC) using a thermal polycondensation method. The synthesized copolymer was characterized using Fourier transform infrared spectroscopy (FT-IR), carbon-13 nuclear magnetic resonance spectroscopy, and differential scanning calorimetry. The ability of CA-co-GLC to inhibit deposition of inorganic scales such as calcium carbonate was investigated under varying temperature and pH medium. The evaluation of inhibition efficiency (IE) was conducted using the static scale inhibition method. The mechanism of the inhibitor’s action was investigated via growth solution analysis, measurement conductivity, and analysis of CaCO_3_ using FT-IR and scanning electron microscopy. The results obtained showed that the CA-co-GLC had good IE at an elevated temperature reaching 75% at 100 °C, pH 7.5, and 10 ppm copolymer dose. Using the same dose, the IE reached 66% at 50 °C and pH 10. The CA-co-GLC did not chelate Ca^2+^ in water, but led to a change in polymorphism, making it brittle and able to slip easily from the surface. Its action principally prevented the adhesion of calcium carbonate onto the surface.

## 1. Introduction

Today, the world is confronting the threatening problem of freshwater shortage, and desalination is viewed as a workable method to mitigate this problem. However, increasing the concentration of dissolved mineral ions up to a critical point of supersaturation in the circulating cooling water system of desalination plants results in the depositing of strongly adherent scales on the surface, e.g., CaCO_3_, BaSO_4_, CaSO_4_, and Ca_3_(PO_4_)_2_ [1]. Scale deposition causes significant economic losses because of low flow rates and increases the temperature and pressure above the allowed limit. Indeed, these damages might lead to complete blockage of pipes and disruption of plant production [2,3,4,5,6].

Existing scale inhibition methods can be divided into two types: physical and chemical. The physical scale inhibition method is considered a promising environmentally friendly means, but it is not sufficiently efficient. It is achieved using certain energy sources such as magnetic and electric energy; in this manner, it is not broadly utilized at present [1,4]. The chemical scale inhibition method, in particular antiscalant additives, is currently the most commonly used method. It is simple to use and causes a change in the chemistry of the water system. At present, the commercial antiscalants are predominantly organic phosphonates, which cause environmental pollution [7,8,9,10]. Therefore, for the time being, with the growing necessity of environmental protection, the innovation of a new environmentally friendly green antiscalant is considered an important research topic.

Anionic polymers that contain carboxylic groups have been considered the most useful as scale inhibitors because they have advantageous features such as non-toxicity, good thermal stability, and a small concentration dose up to ppm [1,2,3,11]. However, these polymers also have some defects; some are expensive and difficult to obtain in large quantities, and others need more development to improve their scale inhibition efficiency under some conditions. Therefore, there is a need to develop an inexpensive, environmentally friendly, and efficient polymeric scale inhibitor.

Hyperbranched polymers are extremely branched structures containing a large number of terminal functional groups. These chains generally have low viscosities and high solubility. The low intrinsic viscosity refers to their packed structure, whereas the high solubility is because of the large number of end functional groups excited per macromolecule [12,13,14].

The objective of this work was the synthesis of a hyperbranched polyester based on citric acid (CA) and glycerol (GLC) via the thermal polycondensation method. CA is a biocompatible and inexpensive organic material. This product is used at a large scale in industrial fields such as agribusiness, pharmaceuticals, and detergents. GLC is the main component in the synthesis of phospholipid and has a multitude of usages in the pharmaceutical, cosmetic, and food industries [15,16]. Accordingly, one can propose that copolymers dependent on CA and GLC should be biocompatible and have unique properties.

In this study, the effects of copolymer citric acid and glycerol (CA-co-GLC) on calcium carbonate deposition were investigated. The inhibition efficiency (IE) was evaluated by measuring the weight of the adherent scale, conductivity measurement, and solution analysis. The effects of temperature, CA-co-GLC dose, and pH on scale efficiency were also studied.

## 2. Materials and Methods

### 2.1. Materials

Citric acid monohydrate (99.5%, MERCK, Darmstadt, Germany, AR grade) (CA), glycerol anhydrous (GLC) (98%, MERCK, Darmstadt, Germany, AR grade), ethylene diamine tetraacetic acid disodium (EDTA.Na_2_) (99.0%, Sigma-Aldrich, St. Louis, MO, USA), tetrahydrofuran (THF) (99.9%, MERCK, Darmstadt, Germany, AR grade), Eriochrome Black-T Indicator (EBT), and borax sodium hydroxide buffer solution were purchased from the Sigma-Aldrich Company (St. Louis, MO, USA). All chemicals were employed without further purification. Distilled and de-ionized water was used throughout all the experiments.

### 2.2. Synthesis of Hyperbranched CA-co-GLC

CA-co-GLC was synthesized via the melting polycondensation route. CA and GLC were mixed at a 1:1 molar ratio in three round flasks equipped with a condenser pipe, vacuum inlet, and drying tube containing anhydrous calcium chloride. The mixture was heated under stirring at the beginning to 110, 130, and 150 °C for 40, 50, and 60 min, respectively. Water produced during the esterification reaction was gradually removed through a vacuum pump and anhydrous calcium chloride tube. The viscous solution obtained was cooled at room temperature and then purified three times via dissolution in THF and precipitation in heptanes. Light brown polymer beads were obtained and then dried under a vacuum for 48 h.

### 2.3. Characterizations of CA-co-GLC

#### 2.3.1. FT-IR Analysis

The FT-IR analysis of the (CA-co-GLC) structure was performed using a Perkin Elmer Spectrum 1000 spectrometer (Perkin ELMER, Billerica, MA, USA). The reflectance spectra of the copolymers and their pure components were collected between 400 and 4000 cm^−1^ without any prior treatment. CA-co-GLC samples were examined as a film prepared by solvent casting.

#### 2.3.2. NMR Analysis

The ^13^C NMR spectra were recorded using a JEOL ECS 400 MHz NMR Spectrometer (Peabody, MA, USA), 2.0 mg of copolymer dissolved in 2.0 mL of deuterated DMSO, and 5 mm NMR tubes.

#### 2.3.3. DSC Analysis

The thermal properties of CA-co-GLC, as well as CA were studied using the DSC method with a Shimadzu DSC 60A (Shimadzu, Kyoto, Japan) device previously calibrated with indium. Between 8 and 10 mg of film (copolymer) or powder (CA) samples were packed in aluminum pans and placed in the DSC cell. The samples were scanned from 25 to 200 °C under a nitrogen atmosphere at a heating rate of 20 °C·min^−1^. The thermal curves obtained showed that the samples did not undergo degradation during the heating process.

The glass transition temperature, Tg, was accurately determined on the thermograms from the midpoint indicating the variation in the heat capacity versus temperature. To remove any traces of volatile compounds incrusted in the polymer matrix, such as solvent, water, and residual monomer, Tg values were taken from the second cycle of the DSC process.

### 2.4. Scale Inhibition Evaluation

The growth solution of CaCO_3_ was prepared by mixing an equal amount of CaCl_2_ and Na_2_CO_3_ in stainless-steel jars and then placing them in a water bath for incubation while maintaining them at a certain temperature for 16 h. The pH of the growth solution was controlled by a NaOH/borax buffer solution. Then, the jars were removed from the water bath, and the growth solution was poured out and allowed to dry. Adherent and accumulated CaCO_3_ scales were weighed accurately by measuring the increased weights of jars. Scale inhibition efficiency (*IE*) was calculated using the following Equation (1):

(1) $$IE\left(wt\%\right)=\frac{\Delta m_{o}-\Delta m_{1}}{\Delta m_{o}}\times 100$$

where Δ*m_o_* and Δ*m*_1_ are the increased weight of the stainless steel in the blank and in the jar containing the antiscalant, respectively.

### 2.5. Determination of Residual Calcium Ion Concentration

The residual calcium ion concentration was evaluated via titration of the growth solutions resulting from filtration after the incubation period and cooling. The calcium ions in the filtrate were then titrated using a 0.01 M aqueous solution of EDTA.Na_2_.

### 2.6. Determination of Conductivity

The CaCl_2_ solution was placed in clean stainless-steel jars and then immersed in a water bath maintained at 25 °C with its pH controlled using the NaOH/borax buffer solution. The sensor of the conductometer (Beta 81, CHK Engineering, Smithfield, Australia) was carefully cleaned by 1M H_2_SO_4_ and then by distilled water to remove all deposits. Portions of the 0.5 mL of Na_2_CO_3_ solution were added using a titration burette to the CaCl_2_ solution under continuous stirring. The conductivity of the resultant media was continuously measured after each addition. The Na_2_CO_3_ solution was constantly added until a sudden decrease in the conductivity occurred. The first low reading in the conductivity refers to the critical point for the deposition of calcium carbonate scale. A comparison of the conductivity of the growth solution was performed in the presence and absence of CA-co-GLC.

### 2.7. Scale Analysis

Under the optimal conditions for inhibition, the crystals of CaCO_3_ were carefully collected and dried in an oven vacuum for 24 h. The SEM micrographs of the surface morphology of the crystals coated with gold were recorded using the scanning electron microscope JEOL JSM 6360 (JEOL, Tokyo, Japan). The crystal structure of CaCO_3_ was analyzed via FT-IR spectroscopy (Spectrum Two, PerkinElmer, Waltham, MA, USA) using KBr pellets as a blank sample.

## 3. Results and Discussion

### 3.1. Synthesis of CA-co-GLC

**Scheme 1:**
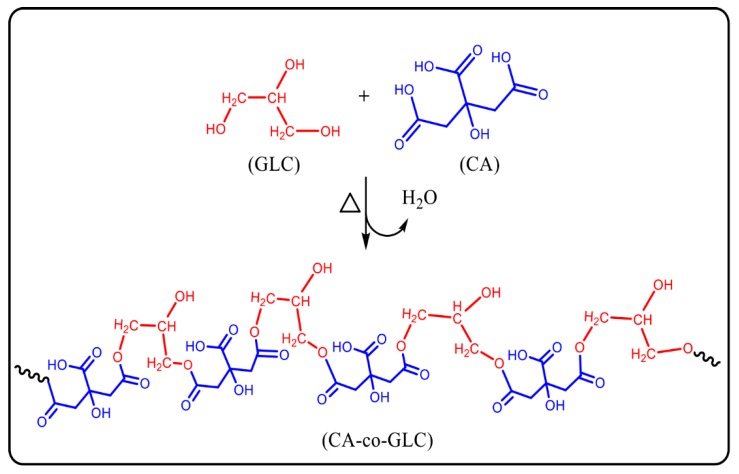
Melting polycondensation reaction of citric acid (CA) and glycerol (GLC) and linear polymer formation.

**Scheme 2:**
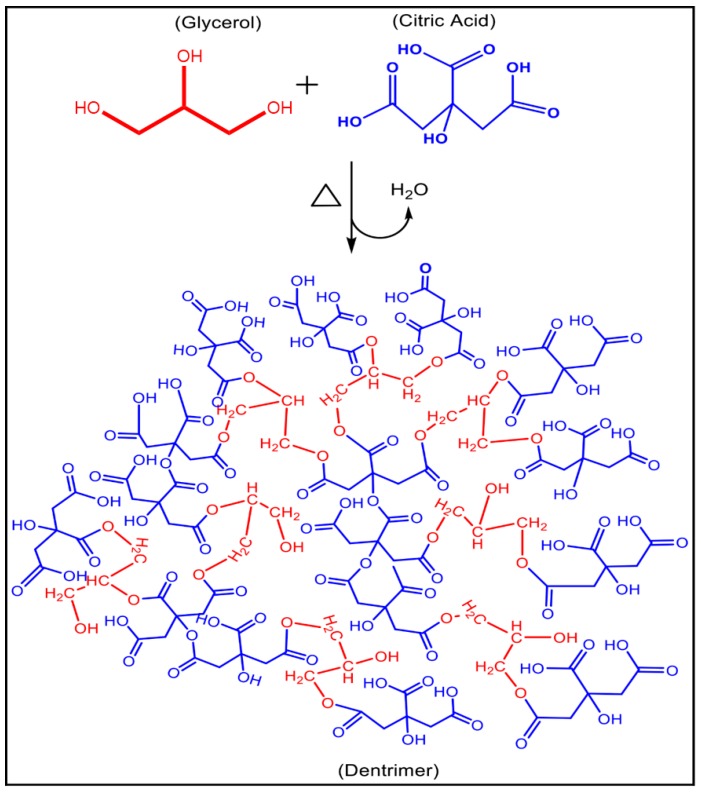
Melting polycondensation reaction of CA with GLC and hyperbranched CA-co-GLC dendrimer formation.

Hyperbranched polyester CA-co-GLC was obtained via the formation of ester bonds between the CA monohydride and GLC monomers through melting polycondensation polymerization. Decomposition of CA before reaching its melting point is a significant problem for the melt polycondensation reaction [17]. However, the use of CA monohydride solves this problem because of its lower melting point (100 °C) compared to that of ordinary CA (153 °C). The citric acid molecule contains three carboxyl groups, as well as one hydroxyl group; thus, there is a high probability to gain a tree-like structure copolymer. According to Adeli and others [12,13,14,17], gradually increasing the temperature of the melting polycondensation reaction leads to an increase in the probability to obtain a hyperbranched polymer. The theoretical speculations of the CA-co-GLC included linear or dendritic polymers as schematized in Scheme 1 and Scheme 2, respectively.

In linear CA-co-GLC, the two carboxyl groups on the secondary atoms of a CA monomer react with the hydroxyl groups on the primary carbon of the GLC monomer. Meanwhile, the carboxyl groups on the tertiary-carbon atom of the CA monomer and the hydroxyl group on the secondary-carbon atom of the GLC remain free without involvement in the esterification reaction (Scheme 1). The second route leads to a dendrimer copolymer in which all three carboxyl groups of the CA react with the three hydroxyl groups of the GLC (Scheme 2).

### 3.2. Characterization of CA-co-GLC

**Figure 1:**
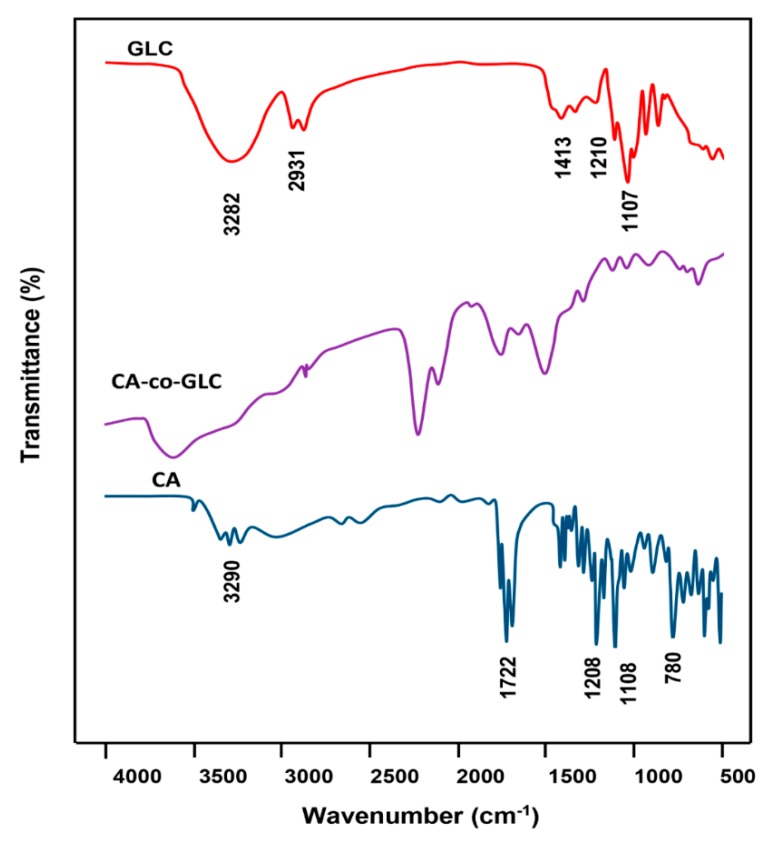
FT-IR spectra of the CA and GLC monomers and CA-co-GLC copolymer.

**Figure 2:**
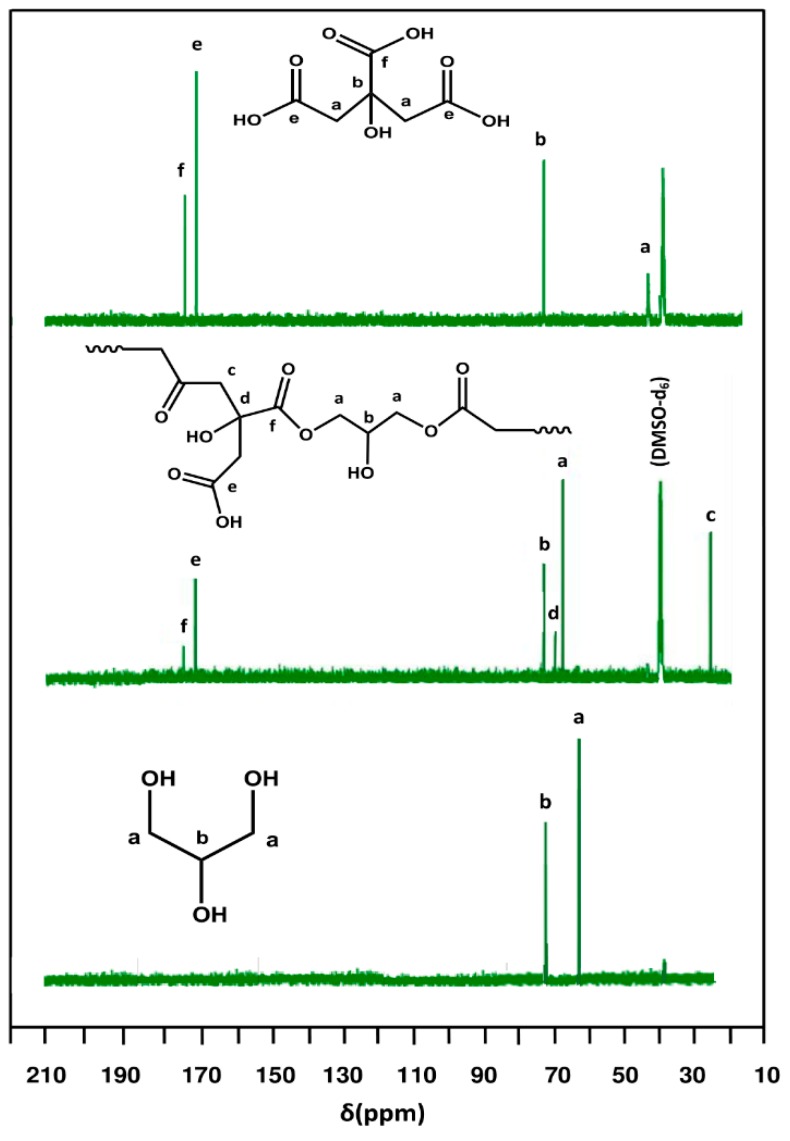
^13^C NMR spectra of CA and CA-co-GLC in DMSO-d6.

The structure of the CA-co-GLC was highlighted by the FT-IR and ^13^C NMR spectroscopy analysis. Indeed, Figure 1 shows the FT-IR spectra of this copolymer and its two pure comonomers CA and GLC. The CA that contained three carboxylic groups showed in its spectrum two characteristic absorption bands at 1648 and 1730 cm^−1^ assigned to the acidic and esteric carbonyl groups, respectively. Meanwhile, the GLC spectrum presented its characteristic peak at 3292 cm^−1^, which was assigned to the three hydroxyl groups. In the CA-co-GLC spectrum, the stretches of C–O–C formed during the reaction were localized at 1222 and 1057 cm^−1^ in which one was stronger and broader than the other, proving the polycondensation of GLC and CA monomers via the esterification reaction. Figure 2 shows the ^13^C NMR spectra of CA, GLC, and their copolymer. Signals (f) and (d) localized at 70.0 and 174 ppm were attributed to the CA monomer units, while those of the carbons (a) and (b) characterizing the GLC monomer units were at 67.0 and 63.2 ppm, respectively. The signal at 171.5 ppm was attributed to the two carbons (e) of the acidic carbonyls of the CA commoner unit and that at 26.0 ppm to the carbon(c) bearing the carboxyl groups of this same unit.

**Figure 3:**
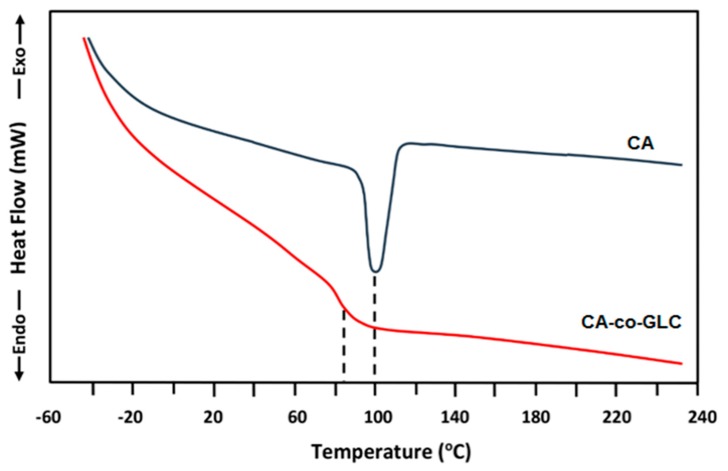
DSC thermograms of pure CA and CA-co-GLC.

The thermal analysis of copolymer and their monomer components carried out by the DSC method showed through the thermograms in Figure 3 a melting temperature for CA at 100 °C, while the thermal curve of CA-co-GLC showed a glass transition temperature at 87.54 °C. No traces of a CA monohydrate melting peak were observed on the thermogram of this copolymer, indicating that the CA monohydrate entirely reacted with GLC to produce the copolymer.

### 3.3. Scale Inhibition Efficiency of CA-co-GLC

#### 3.3.1. Effect of CA-co-GLC Concentration

**Figure 4:**
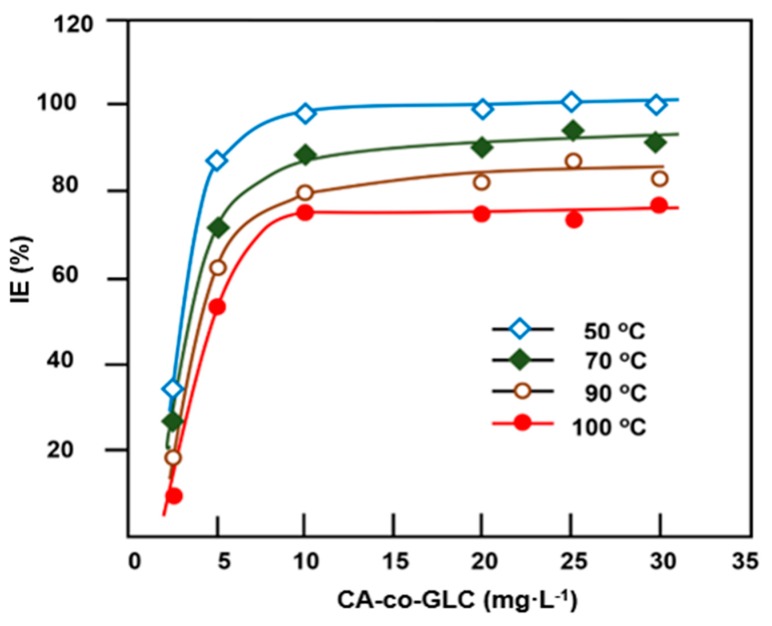
Variation in the scale inhibition efficiency (IE) versus the concentration of CA-co- GLC at a media pH of 7.5 and with 1000 mg·L^−1^ Ca^2+^.

The change in the scale IE versus the concentration of CA-co-GLC was assessed at 50, 70, 90, and 100 °C; the results obtained are shown in Figure 4. As can be seen from these curve profiles, at any temperature investigated, the IE dramatically increased when the concentration of CA-co-GLC was less than 6.0 mg·L^−1^, but stabilized as this was exceeded. It was also shown that the scale IE was practically selective (98 wt% and greater) at 50 °C at a concentration greater than 10.0 mg·L^−1^. The IE minimum (74 wt%) was reached when the temperature was 100 °C, at which an increase in the temperature decreased the efficiency by greater than 24 wt%.

#### 3.3.2. Effect of Temperature

**Figure 5:**
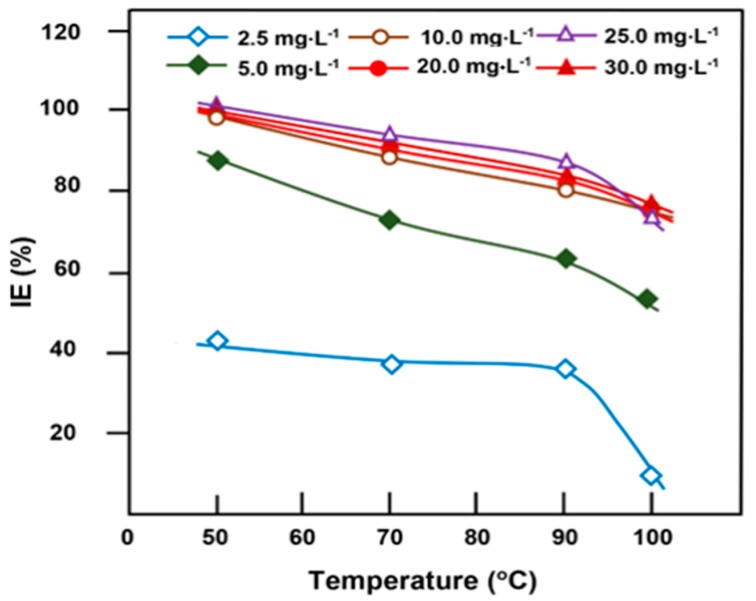
Variation in the scale IE versus the temperature in the media at a pH of 7.5 and 1000 mg·L^−1^ Ca^2+^.

The variation in the scale IE versus the temperature of media was assesses at different concentrations of inhibitor; the results obtained are shown in Figure 5. As can be seen, the scale IE was significantly affected by the temperature. Indeed, these curve profiles showed a decrease in the scale inhibition performance as the temperature increased, and this became more prominent when the temperature exceed 80 °C, notably at low CA-co-GLC concentrations in the media. The decrease in the scale IE as the temperature increased above 80 °C seemed to be because of two phenomena: (i) A higher temperature led to a reduction of the adsorption capacity of the crystal nucleus and improved the desorption process. In this situation, the growth rate of the crystals will be accelerated, and the crystals aggregated to form larger particles. (ii) The second one was that the reverse solubility of CaCO_3_ decreased with increasing temperature [10,18]. Finally, the CA-co-GLC featured the best heat resistance and could achieve satisfactory scale inhibition in water supply systems with a significant temperature change. A similar finding was also observed by Li et al. [19] using six types of scale inhibitors on CaCO_3_ such as the scale inhibitor SQ1211 (Shandong Tian Qing Science and Technology Development Co., Ltd., Beijing, China); scale inhibitors 190 and 265 (Nalco Company, Beijing, China); and LinHai-4, LinHai-1, and LinHai-3 (Shandong LinHai Science and Technology Co., Ltd., Beijing, China). These authors explained this by a decrease in the solubility of CaCO_3_ with increasing temperature. Indeed, this phenomenon is well known for this type of salt. In addition, the scale layer more quickly formed and thickened as the temperature increased, which can also reduce the scale IE.

#### 3.3.3. Effect of the pH of the Media

**Figure 6:**
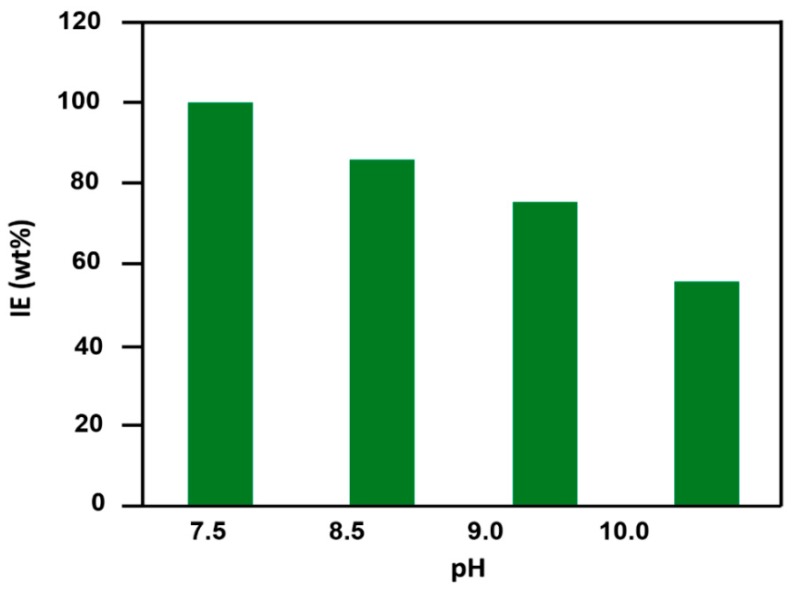
Influence of the pH of the media on the scale IE at 50 °C for a concentration of 10 mg·L^−1^ of CA-co-GLC and 1000 mg·L^−1^ of Ca^2+^.

The influence of the pH of the media on IE was assessed at 50 °C with a concentration of 10 mg·L^−1^ of CA-co-GLC by varying the pH of the media from 7.5 to 10. The data obtained, shown as a diagram in Figure 6, showed an important decrease in the scale IE when the pH of the media increased. Indeed, a practically 100% IE was reached at pH 7.5, but decreased by nearly one-half when the pH of the media was 10.

With an increase in the pH of the media, the hydroxyl ion (OH^−^) concentration increased and thereby the equilibrium of the reaction between OH^−^ and HCO_3_^−^ shifted toward the production of CO_3_^2−^. In this manner, this process favored the precipitation of CaCO_3_, and consequently, the scale IE decreased [20]. The results obtained for the behavior CA-co-GLC in alkaline media agreed with [21], which used acrylonitrile-acrylic acid (AN-AA) and acrylonitrile-methacrylic acid (AN-MAA) copolymers as the inhibitors of the deposition of CaCO_3_. With an increased pH above to 7.5, the efficiency reached 30% and 50% for (AN-AA) and (AN-MAA), respectively, at pH 8.5 and 50 °C. This decrease in efficiency was attributed to the copolymers acting as coagulants due to the flocculating nature of alkaline media [21,22,23]. The same phenomenon was observed by Senthilmurugan and his companions [22] when they used maleic acid-acrylic acid (MA-AA) and maleic acid-acrylamide (MA-AAD) copolymers to inhibit the deposition of calcium sulfate, and the efficiency did not exceed 25%. A blend of poly(aspartic acid-co-citric acid) and polymaleic acid (PAC-HPMA) was prepared and used to inhibit calcium phosphate scale [24]. The (PAC-HPMA) showed higher alkaline tolerance since the inhibition efficiency was still 60.2% at pH 11 and 80 °C. In addition, the carboxylic acid groups were unstable in alkaline media because their ionization degree increased, and only the carbonyl group from the total protonated scale inhibitor with carboxyl groups could form hydrogen bonding with water from the surface of insoluble salt nuclei [19]. As a result, the effectiveness of the scale inhibition decreased when the pH increased from 7.5 to 10.

### 3.4. Solution Analysis

#### 3.4.1. Determination of Residual Calcium Ion Concentration

**Scheme 3:**
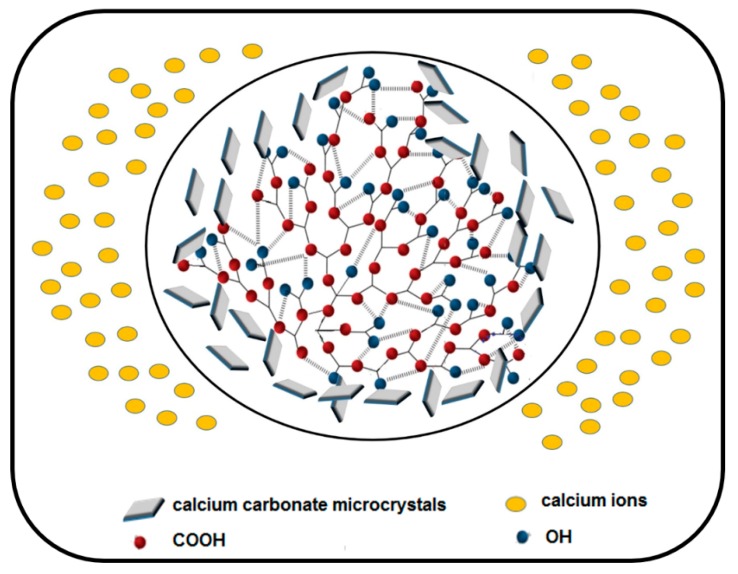
Inhibition mechanism of CA-co-GLC.

Based on the mechanism of scale inhibitors, there are two paths that are considered as the fundamental goals behind their use. In the first path, scale inhibitors delay the appearance of CaCO_3_ crystals out of bulk solution; however, this leads to an extended incubation period that is as long as possible, and they deform the crystals by clogging the ions of the correct disposition during crystal growth, leading to fragile crystals that are unable to adhere strongly to the surface [4,25]. The second path is preventing nucleation by complexing calcium ions through functional groups on inhibitors; thus, the solubility of calcium carbonate in water increases [4,26]. Because there are no findings in the literature studying the scale inhibition mechanism of hyperbranched polyester, the investigation relied on evaluating the effect of CA-co-GLC doses on the concentration of residual Ca^2+^, and the results are presented in Table 1. As can be seen, the concentration of residual Ca^2+^ did not significantly change with increasing CA-co-GLC concentration. This contradicts the inhibition taking place according chelate free Ca^2+^ ions. From the CaCO_3_ analysis by FT-IR and SEM images, Figures 12 and 13 indicate the change in polymorphic distribution from calcite to aragonite. Therefore, the inhibition mechanics occurred by the change in the crystals’ growth. The chelating of Ca^2+^ did not occur, which could be caused by the hindrance of carboxylic groups by the hydrogen bonds, as schematized in Scheme 3.

These results differed from some previous studies [10,19,23,27,28,29] indicating that the main mechanism of antiscalant inhibition is attributed to the uptake of Ca^2+^ free ions. On the other hand, the study submitted by [30] indicated that carboxylic acids (acrylic acid, maleic acid, tartaric acid, malic acid, succinic acid, and citric acid) were not affected to the nucleation of CaCO_3_ crystals, but crystal growth was changed by the adsorption of antiscalants on the surface of the CaCO_3_ crystals. These phenomena were clarified by assuming a stronger affinity of the carboxylic acids for CaCO_3_ microcrystals than for the free Ca^2+^ ions in solution, thus consistent with the behavior of CA-co-GLC in this report.

#### 3.4.2. Determination of the Conductivity

**Figure 7:**
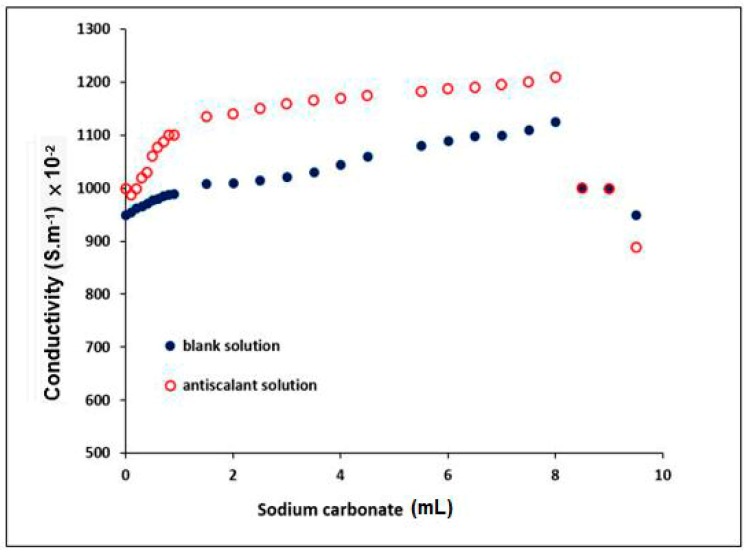
Variation in the conductivity in the presence and absence of 10 mg L^−1^ of CA-co-GLC at 25 °C and pH 7.5.

**Figure 8:**
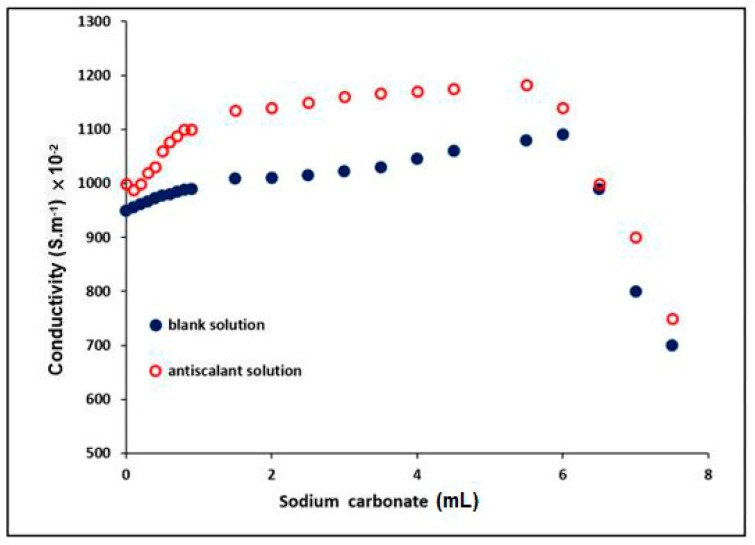
Variation in the conductivity in the presence and absence of 10 mg·L^−1^ CA-co-GLC at 25 °C and pH 8.5.

**Figure 9:**
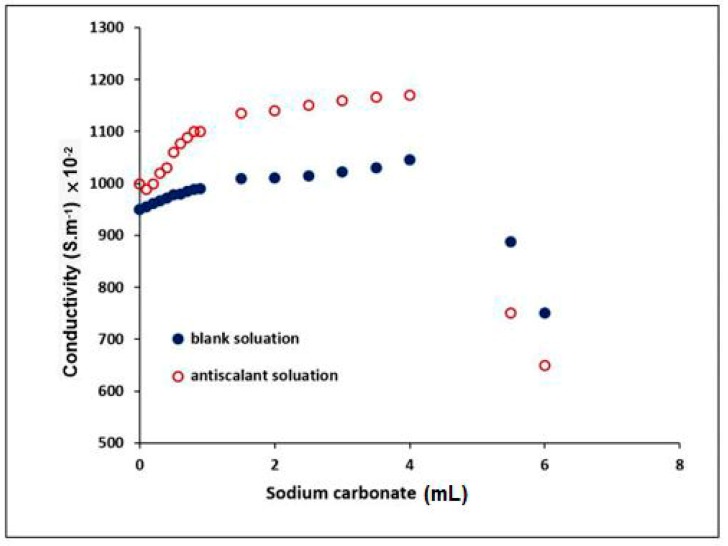
Variation in the conductivity in the presence and absence of 10 mg·L^−1^ CA-co-GLC at 25 °C and pH 9.

**Figure 10:**
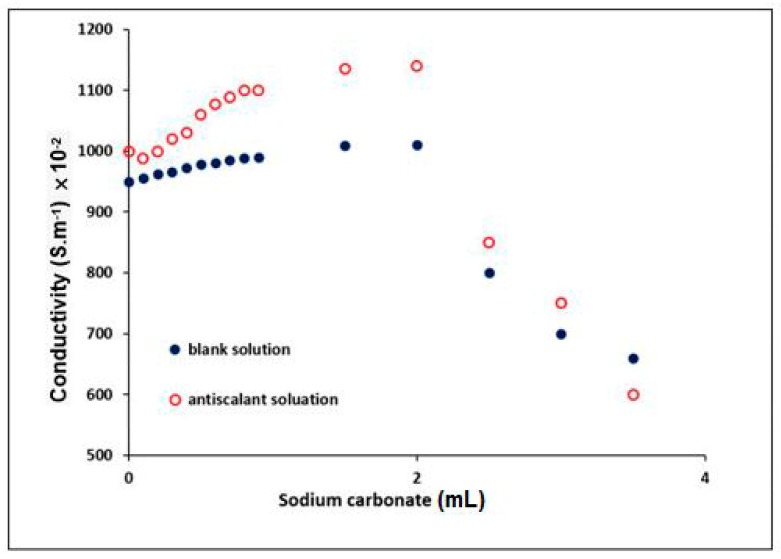
Variation in the conductivity in the presence and absence of 10 mg·L^−1^ CA-co-GLC at 25 °C and pH 10.

**Figure 11:**
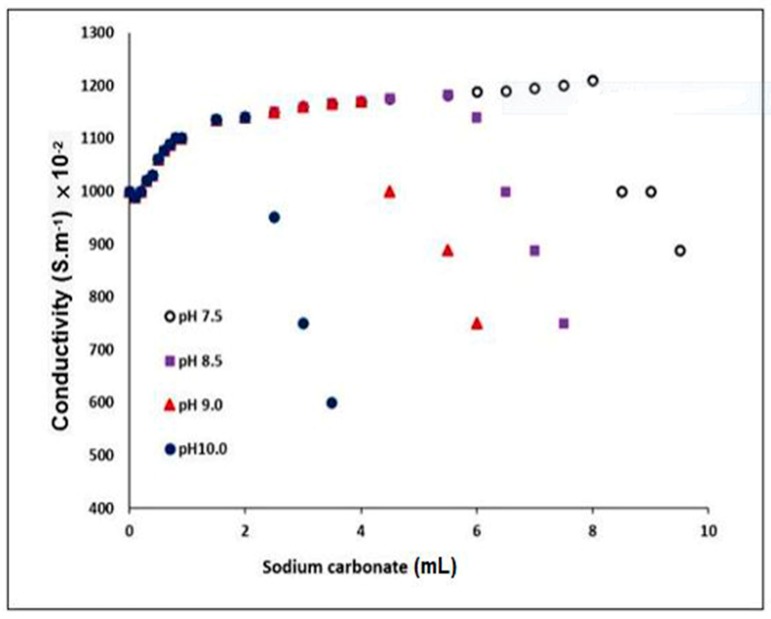
Variation in the conductivity in the presence of 10 mg·L^−1^ of CA-co-GLC at 25 °C and in different pH of the media.

Figure 7, Figure 8, Figure 9, Figure 10 and Figure 11 show the change in the conductivity of the growth solution in different pH of the media at 25 °C with gradual additions of Na_2_CO_3_ and 10 mg·L^−1^ of CA-co-GLC. The conductivity continuously increased with increasing Na_2_CO_3_. When the solution reached the critical point of supersaturation, the conductivity reached its highest value. An excess amount of Na_2_CO_3_ caused the onset of nucleation and deposition of CaCO_3_; therefore, the conductivity of the solution decreased because of the lack of soluble ions. It is very important to note that the critical point of supersaturation for all pH of the media in the presence of CA-co-GLC did not substantially change compared to the blank solutions. Therefore, CA-co-GLC did not appreciably change the supersaturation point with respect to the blank solution. In other words, CA-co-GLC did not chelate calcium ions, so for the Ca^2+^ ion, the concentration remained unchanged. These results agreed well with those presented in Table 1, in which the residual Ca^2+^ concentration did not change after adding CA-co-GLC. This result supports that the inhibition mechanism of CA-co-GLC occurs only through the distortion of crystals.

The supersaturation critical point obtained for different pH of the media is shown in Figure 7, Figure 8, Figure 9 and Figure 10, and the results are summarized in Figure 11. These data show that a stronger alkali medium leads to accelerated emergence of the critical point of supersaturation because of the reduced incubation period and earlier CaCO_3_ deposition. The principle explanation of these findings is the increase in the OH^−^ concentration that led to a shift in the chemical balance to the right, causing the increase of the CO_3_^−^ concentration, thus increasing the CaCO_3_ deposition according to Equations (2) and (3) [20].

HCO_3_^−^ + OH^−^ ⇌ CO_3_^2−^ + H_2_O (2)

Ca^2+^ + CO_3_^2−^ ⇌ CaCO_3_ (s) (3)

#### 3.4.3. Calcium Carbonate Crystal Analysis

**Figure 12:**
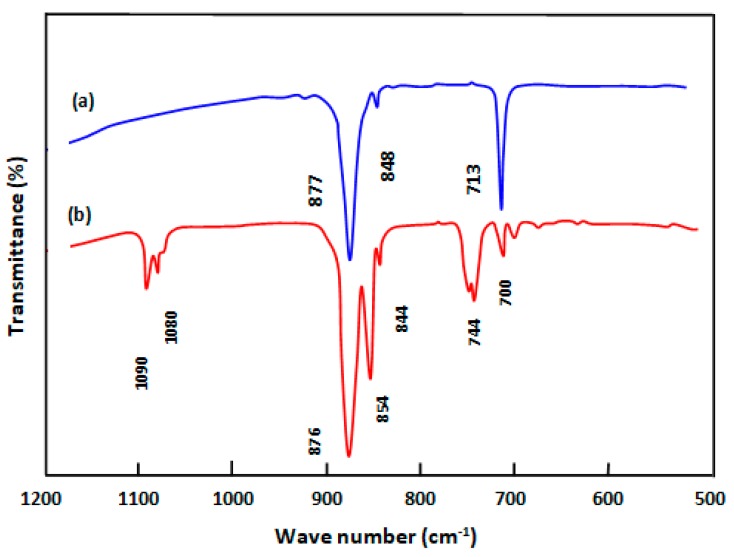
FT-IR spectra of the calcium carbonate crystals formed in the blank (**a**) and in 10 mg·L^−1^ CA-co-GLC (**b**).

The CaCO_3_ crystals were analyzed using the FT-IR method through a comparison of the spectrum of the calcium carbonate crystals formed in the blank solution and that of the other form obtained in the presence of 10 mg·L^−1^ CA-co-GLC as shown in Figure 12a,b, respectively. The CaCO_3_ crystals created in the blank had the characteristic absorption peak of calcite at 713 cm^−1^ and 877 cm^−1^, which corresponds to the vibrations of OCO out-of-plane and OCO bending in-plane deformations in CO_3_^2−^, respectively [31]. For the crystals created in the presence of CA-co-GLC, the peaks of the calcite absorption, which are typically observed at 713 and 877 cm^−1^, nearly disappeared, and the characteristic absorption peaks of aragonites were observed at 700 cm^−1^, 854 cm^−1^, and 1080 cm^−1^ [32].

The FTIR spectrum shown in Figure 12 confirms the deformation of CaCO_3_ crystals caused by the CA-co-GLC because of the conversion of calcite to aragonite.

**Figure 13:**
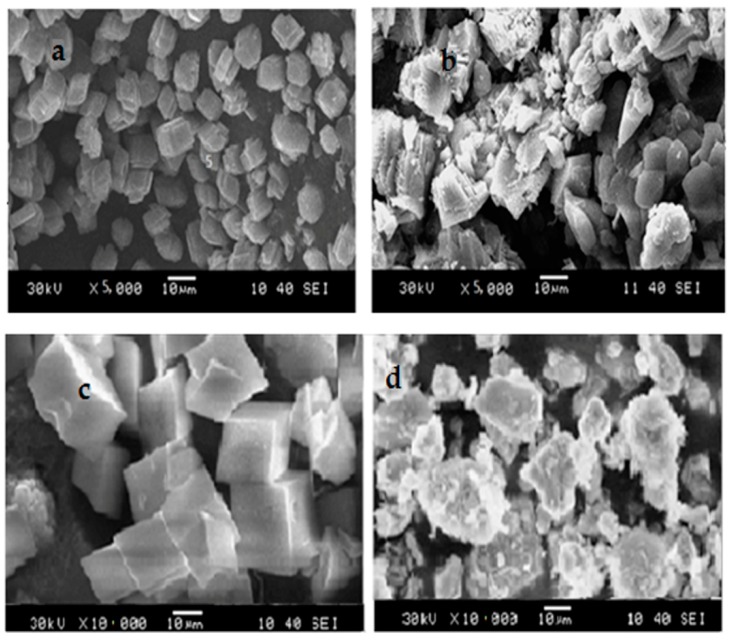
SEM images at different resolutions of the CaCO_3_ scale (**a**,**c**) in the blank solution and (**b**,**d**) in the presence of CA-co-GLC (Ca^2+^ 1000 mg·L^−1^, pH 7.5, T 50 °C).

Figure 13a–d shows that the CaCO_3_ formed in the blank (a,c) had compact crystals with a smooth and glossy surface. Their cubic and regular shape was quite clear with the rhombohedral structure of calcite. In the presence of 10 mg·L^−1^ of CA-co-GLC (b,d), the crystals obviously changed. They lost their edges and converted to stacked shapes, and their surface became rough and curvy.

The laboratory viewing confirmed this fact. The inhibited CaCO_3_ crystals seemed to be fragile and fluffy crystals similar to cotton and therefore easily slipped and did not adhere to the surface; this is what distinguished the aragonite crystals. The crystals of CaCO_3_ created in the virgin solution strongly stuck on the surface and required the use of a strong acid for cleansing the surface. This distinguished the cubic and regular shape of the calcite morphology.

Based on the above, the mechanism was clarified; the change of the cubic and regular shape of CaCO_3_ crystals (calcite polymorph) after adding CA-co-GLC took place through the adsorption of the chains on CaCO_3_ microcrystals, thus affecting the crystals’ growth, which caused a change in the polymorphic distribution of aragonite crystals. These results agreed with those of the literature such as [8], which indicated that the CaCO_3_ crystals generated in the blank comprised calcite, and this polymorph completely changed, thus forming a vaterite crystal after adding modified collagens as antiscalants; the work in [33] also analyzed the CaCO_3_ crystals after treatment with polyaspartic acid; the calcite crystals were the main form in the blank, but the CaCO_3_ crystals became a mixture of vaterite and aragonite crystals after polyaspartic acid addition. The same phenomenon was repeated in [20] that CaCO_3_ crystals had changed from calcite to aragonite after adding commercial scale inhibitor SQ-1211 (Shandong TianQing Science and Technology Development Co., Ltd., Beijing, China). 

## 4. Conclusions

In conclusion, the CA-co-GLC polyester was easily synthesized by combining CA with GLC via a melt polycondensation pathway at a progressive temperature up to 150 °C. It was also shown that this copolymer in very small amounts (10 ppm) was capable of inhibiting CaCO_3_ scale formation and had an excellent scale inhibiting effect in high temperate and alkaline media, up to 75% at 100 °C in a medium of pH 7.5 and reaching 66% at 50 °C in a medium of pH 10. It was also found that the mechanism of inhibition of CA-co-GLC differed from that of other scale inhibitors. That it did not chelate Ca^2+^ was proven by the equal Ca^2+^ concentration of in the blank solution and the solution containing CA-co-GLC. The critical conductivity was also unchanged by CA-co-GLC. The inhibition mechanism of CA-co-GLC was based on only the change in polymorphism of CaCO_3_ crystals from calcite to aragonite. Nucleation of CaCO_3_ crystals was not influenced by the presence of CA-co-GLC because the chelation of free Ca^2+^ ions in solution did not take place here. Despite the chains of CA-co-GLC containing a huge number of carboxylic groups, they could not trap free Ca^2+^ ions, and this was due to the presence of an intensive amount of hydrogen bonds between the polymeric chains. Subsequently, the inhibition mechanism was highlighted by assuming a stronger affinity of the CA-co-GLC for CaCO_3_ microcrystals than for the free Ca^2+^ ions in solution. The change in the polymorphic distribution of CaCO_3_ from calcite to aragonite was the main action of CA-co-GLC, resulting in the CaCO_3_ slight sticking to the surface.

## Figures and Tables

**Table 1:** Concentration of residual Ca^2+^ after the incubation period at different CA-co-GLC concentrations.

Antiscalant Dose (mg·L^−1^)	Residual Ca^2+^ (mg·L^−1^)
0	1000
5	990
10	995
15	994
20	996
30	994
40	993
50	998
